# Long-acting reversible contraceptive use in the post-abortion period among women seeking abortion in mainland China: intentions and barriers

**DOI:** 10.1186/s12978-018-0543-2

**Published:** 2018-05-24

**Authors:** Zhongchen Luo, Lingling Gao, Ronald Anguzu, Juanjuan Zhao

**Affiliations:** 1 Nursing Department, Guizhou Provincial People’s Hospital, Guiyang, China; 2 School of Nursing, Sun Yat-sen University, 74#, Zhongshan Road II, Guangzhou, 510089 China; 3 School of Public Health, Makerere University, Kampala city, Uganda

**Keywords:** Long acting reversible contraception, Intrauterine devices, Implants, Induced abortion, Intention, Barriers

## Abstract

**Background:**

This study aimed to describe the intentions of and barriers to the use of long-acting reversible contraceptives (LARCs) in the post-abortion period among women seeking abortion in mainland China.

**Methods:**

A cross-sectional study was conducted from July 2015 to December 2015 using a waiting room questionnaire. A total of 381 women seeking abortions were recruited at a public hospital abortion clinic. The outcome variable was an ‘intention-to-use’ LARCs in the immediate post-abortion period. Chi-square tests were used to assess associations between categorical variables. Statistically significant variables (*p* ≤ 0.05) were then further analyzed by logistic regression.

**Results:**

Among 381 respondents, 42.5% intended to use LARCs in the immediate post-abortion period; 35.2% intended to use intra-uterine devices (IUDs); and 13.9% intended to use implants. Previous use of LARC was a predictor for an intention to use LARCs (odds ratio [OR] = 2.41; 95% confidence interval [CI]: 1.06–5.47). Participants with one or no child had reduced odds for an intention to use LARC (OR = 0.32, 95% CI: 0.15–0.47 and OR = 0.29, 95% CI: 0.13–0.68, respectively). Women with a higher sex frequency (at least once per week) showed increased odds for LARC use (OR = 3.34; 95% CI: 1.03–10.78) and married women were more likely to use LARC than single women (OR = 1.57; 95% CI:1.00–2.47). Women who planned to have another baby within two years were more likely not to use LARCs in the immediate post-abortion period (OR = 0.97; 95% CI: 0.43–2.12). Barriers to the use of LARCs were anxiety relating to impaired future fertility (56.2%), LARCs being harmful to health (45.2%), irregular bleeding (44.3%), risk of IUD failure (41.6%) and lack of awareness with respect to LARCs (36.1%).

**Conclusions:**

Intention to use LARCs was predicted by marital status, frequency of sexual activity, number of children, planned timing of next pregnancy, and previous LARC use. Impaired future fertility, being harmful to health, irregular bleeding, risk of complications, and lack of awareness with regards to LARCs were the main barriers in their potential use.

## Plain English summary

Long-acting reversible contraceptives (LARCs) are the most cost-effective contraceptives for the prevention of unintended pregnancies, and the use of these medications during the post-abortion period reduces the rate of repeat abortions. However, the willingness of women to use LARCs during the post-abortion period remains uncertain; intentions and potential barriers to their use therefore require further investigation. This study explores the intentions and barriers to the use of LARC in the post-abortion period among pregnant women seeking abortion in mainland China.

Information was collected using a field questionnaire survey by convenience sampling methods. The survey contained 18 questions including socio-demographic information, sexual and reproductive health history, awareness, experience, intention, and barriers to use for each type of LARC in the immediate post-abortion period.

Of the 381 women seeking abortion with unintended pregnancy, approximately 42.5% were interested in using LARCs during the post-abortion period. 35.2% intended to use intra-uterine device (IUDs), while nearly 13.9% of the women intended to use the implant method during the post-abortion period, which suggested that this cohort of women preferred IUDs over implants. Intention to use LARCs was predicted by marital status, frequency of sexual activity, number of children, planned timing of the next pregnancy, and previous LARC use. Impaired future fertility, being harmful to health, irregular bleeding, risk of complications, and lack of awareness were the main barriers to the use of LARC. In order to increase access to LARCs in order to reduce unintended pregnancies in women, education and communication should focus on alleviating barriers to the use of LARCs.

## Background

Unintended pregnancy is an important public health challenge cross the world, as well as in mainland China. Unintended pregnancy has negative effects on the health of the mother and baby, leading to a sizeable, and potentially avoidable, cost burden [1]. According to the Chinese ‘health and family planning statistics annual publication’ (2014), unintended pregnancy results in 6.62 million abortions in mainland China [2]. Moreover, the rate of repeat induced abortions due to unintended pregnancies is also high in mainland China, with nearly half of the women seeking abortion having had at least one previous induced abortion [3]. Unintended pregnancy usually occurs due to contraceptive issues including emergency contraception failure (33.1%), incorrect use of contraception or a lack of condoms (28.6%) and use of the rhythm method (21.9%). Short-term family planning methods are less effective, and have a higher risk of contraceptive failure [4].

Long-acting reversible contraceptives (LARCs) are a type of contraceptive that is easily reversed and do not rely on the user to maintain efficacy for at least three years. Typical examples include intra-uterine devices (IUDs) and implants [5]. These are user-independent and exhibit higher compliance than short-acting methods [6]. Almost all women of reproductive-age who use LARCs can return to a fertile state, as the effects are reversible [5]. The mean time until return to fertility following the use of implants or IUDs is within days of cessation; faster than all progestogen-only oral contraceptives [5]. Trussell and Hassan et al. conducted a study to estimate the relative costs of short-acting reversible methods and LARC methods, and reported that LARCs were more cost-effective than short-acting reversible methods such as oral contraceptives, the ring, the patch, or injections over a three-year period [7]. The American College of Obstetricians recommends LARCs as an effective method with which to reduce the risk of unintended pregnancy and abortion [8].

Previous evidence has also shown that the use of LARCs during the post-abortion period reduces repeat abortion rates [9, 10]. However, LARC methods have been under-utilized during the immediate post-abortion period in mainland China [11, 12]. To provide a wider choice of more effective methods, and to encourage the continuation of this method, it is important to understand the intentions and barriers to LARC use in women seeking abortion, so that effective counseling practices and promotional policies can be implemented. However, little is known about the intentions and barriers to LARC use in Chinese women seeking abortion. Thus, the present study explored the intentions for LARC use and the factors associated with contraceptive choices among women with unintended pregnancy and seeking abortion in mainland China. Barriers to LARC use in the immediate post-abortion period were also identified.

## Methods

### Study design and setting

A cross-sectional study was carried out between July and December 2015 in Guangzhou, a sub-provincial city located in Southeastern China. Guangzhou is the capital of the Guangdong Province, and has a population of approximately 16 million [13]. In Guangzhou, the sex ratio is 109.5 male for every 100 female; 68.6% of women in this region are of reproductive age (15–50 years old) [14]. The total number of health care institutions in Guangzhou is approximately 3749, including 165 hospitals and 16 maternity and child-care centers,. These institutions provide very similar family planning services [15] which are guided by the Post-abortion Care Consortium in May 2002 [16]. One hospital was randomly selected for the present study on the basis that it would provide a sufficient sample size.

The study hospital was A-level, has over 3000 beds and provides leading services for post-abortion care in mainland China. The eligible criteria for inclusion in the study were women seeking artificial abortion due to an unintended pregnancy and not having a diagnosed cognitive impairment. Participants received no incentives for participation in this study. Sample size was calculated using the modified Kish-Leslie formula [17].; this calculation told us that we required a sample of 363 women, based on 95% confidence intervals, 5% precision and the 38.2% prevalence of induced abortion in Guangzhou [18].

### Data collection

Ethical approval was obtained from the study hospital. A trained research assistant (RA), who was a masters student majoring in Nursing, was responsible for collecting data. All eligible subjects who were waiting for their appointments at the abortion clinic of the study hospital were invited to participate in the study. The RA explained details of the purpose and procedure of the study to the women. All women who provided informed written consent were asked to fill in questionnaires in an interview room. The RA remained in the vicinity to answer questions and personally collect the returned questionnaires. A total of 399 women were approached, of whom 381 completed the questionnaires.

### Measures

The immediate post-abortion period was defined as the period of time after undergoing medical or surgical abortion, but before resuming intercourse. LARC referred to the use of either implants or IUD contraceptives.

An 18-item questionnaire was used to collect data. Firstly, the definition of “*intention to use a LARC”* was described as “*intending to use either IUDs or implants in the immediate post-abortion period”*. Subsequently, women were asked whether they intended to use a LARC (Yes or No). Typical questions were *“Would you like to use an intra-uterine device for contraception in the immediate post-abortion period?*” and *“Would you like to use an implant for contraception in the immediate post-abortion period?*”. In addition, one question investigated personal barriers to the use of LARC. Women were asked to select as many factors as possible from a list of 20 that they perceived as barriers to their use of a LARC. In addition, 5 questions were included which pertained to socio-demographic characteristics including age, marital status, educational level, monthly income and occupation. Six questions referred to sexual and reproductive health history, and subjects were asked questions relating to intercourse frequency, contraceptive use, previous abortions, time from the latest abortion until present, number of children, and expected timing of the next pregnancy. Four questions also referred to awareness and experience regarding the use of any type of LARC.

The 18-item survey questionnaire was based on a comprehensive review of the literature relating to LARCs [19–25]. After the questionnaire was developed, it was reviewed for content validity by four obstetricians and five midwives. These experts provided written feedback on the clarity and relevance of the questionnaire but were not requested to provide a quantitative rating for each item. No item was revised. Before the investigation, the questionnaire was piloted with 10 women seeking abortion in this hospital. All women could understand and complete the questionnaire easily, so no changes were required. The 4-week test–retest correlation for the questionnaire was 0.93.

### Statistical analyses

The Statistical Package for Social Sciences (SPSS) v20.0 was used for all data entry and analyses. Descriptive statistics summarized the socio-demographic characteristics of the participants, as well as sexual and reproductive health histories, awareness, experience and intention to use LARCs. Differences in the rate of the women’s intention to use LARCs among groups with different socio-demographic and reproductive characteristics were computed using the chi-square test. Variables identified as being significant (*p* < 0.05) in these initial tests were then entered into a logistic regression model to determine predictors of the women’s intention to use a LARC in the post-abortion period.

## Results

### Socio-demographic characteristics

Table 1 presents the demographic information of our participants. Almost all women (95.5%) were of Han ancestry with a mean age of 26.9 ± 5.7 years. More than half of the participants (55.6%) had at least a junior college education. The majority (85.0%) were employed and approximately half (50.4%) were single, had personal monthly incomes over 4000 RMB (60.9%), and had intercourse at least once a week (61.9%). Nearly half of the participants (45.1%) had a previous induced abortion, about one-third (28.3%) had a one-year interval from the latest abortion until present, 44.6% had at least one child, and 47.5% of the participants did not want any further children, at least for the next two years.

**Table 1:** Socio-demographic and reproductive characteristics of participants (*n* = 381)

Variable	Frequency *f* (%)
Age (years)^a^	
16–19	20 (5.2)
20–29	254 (66.7)
30–44	107 (28.1)
Marital status	
Married	189 (49.6)
Single	192 (50.4)
Level of education	
Junior high school or below	79 (20.7)
Middle special / senior high school	90 (23.6)
Junior college	111 (29.1)
College graduate or higher	101 (26.5)
Monthly income	
<4000 ¥	149 (39.1)
4000–7999 ¥	196 (51.4)
≥ 8000 ¥	36 (9.5)
Occupation	
Unemployed	57 (15.0)
Employed	324 (85.0)
Reproductive characteristics	
Frequency of intercourse	
At least one time per week	236 (61.9)
< 4 times per month	125 (32.8)
< 1 time per month	20 (5.2)
Have used contraception	
No	25 (6.6)
Yes	227 (93.4)
Have previously had an abortion	
No	207 (54.8)
Yes	172(45.7)
Interval between last two abortions (*n* = 172, months)	
< 6 months	23 (13.4)
6–11 months	26 (15.1)
≥ 12 months	123 (71.5)
Number of children	
0	211 (55.4)
1	111 (29.1)
≥ 2	59 (15.5)
Expected timing of next pregnancy (years)	
< 2 years	160 (42.0)
≥ 2 years	118 (31.0)
Do not want more children	63 (16.5)
Unknown	40 (10.5)

^a^Mean (SD) = 26.87 (5.70) years

### Awareness, experience and intention to use a LARC

The majority of participants (93.4%) had previous contraceptive experiences. Although the majority of women (88.7%) were aware of LARCs, few (8.4%) had previous experience using a LARC (7.8% had used IUDs and 1.1% had used implants). Less than half of the women (42.5%) intended to use a LARC during the post-abortion period, with 35.2 and 13.9% intending to use IUDs and implants, respectively (Table 2).

**Table 2:** Relationship with the use of LARCs during the immediate post-abortion period among Chinese women with unintended pregnancy seeking abortions (*n* = 381)

Item	Frequency *f* (%)
Are aware of LARC	
No	43 (11.3)
Yes	338 (88.7)
IUDs	338 (88.7)
Implants	50 (13.9)
Implants and IUDs	50 (13.9)
Previous LARC use	
No	349 (91.6)
Yes	32 (8.4)
IUDs	28 (7.8)
Implants	4 (1.1)
Intended to use LARC in immediate post-abortion period	
No	219 (57.5)
Yes	162 (42.5)
IUDs	134 (35.2)
Implants	53 (13.9)
Implants or IUDs	25 (6.6)

### Predicting the intention of Chinese women to use a LARC

Women were more likely to intend to use a LARC during the post-abortion period if they were married, had a greater frequency of sexual activity, had more than one child, had not planned pregnancy within 2 years or had previously used a LARC (χ^2^ = 11.89, *p <* 0.01; χ^2^ = 6.63, *p* = 0.04; χ^2^ = 20.87, *p <* 0.01; χ^2^ = 11.83, *p <* 0.01; χ^2^ = 7.63, *p <* 0.01) (Table 3).

**Table 3:** Associations between demographic/reproductive factors and an intention to use LARCs within women with unintended pregnancy during the post-abortion period (*n* = 381)

	Intended to use LARC (*n* = 162) *f (%)*	No intended to use LARC (*n* = 219) *f (%)*	*χ* ^*2*^	*P*
Age (years)			4.02	0.13
16–19	7 (4.3)	13 (5.9)		
20–29	101 (62.3)	153 (69.9)		
30–44	54 (33.3)	53 (24.2)		
Marital status			11.89	0.01**
Married	97 (59.9)	92 (42.0)		
Single	65 (40.1)	127 (58.0)		
Level of education			3.40	0.26
Junior high school or below	41 (25.3)	38 (17.4)		
Middle special/senior high school	38 (23.5)	52 (23.7)		
Junior college	42 (25.9)	69 (31.5)		
College graduate or higher	41 (25.3)	60 (27.4)		
Personal monthly income (RMB)			0.12	0.94
< 4000	65 (40.1)	84 (38.4)		
4000–7999	82 (50.6)	114 (52.1)		
≥ 8000	15 (9.3)	21 (9.6)		
Occupation			1.20	0.27
Unemployed	28 (17.3)	29 (13.2)		
Employed	134 (82.7)	190 (86.8)		
Reproductive characteristics				
Frequency of sexual activity			6.63	0.04*
At least once a week	110 (67.9)	126 (57.5)		
< 4 times per month	48 (29.6)	77 (35.2)		
< 1 time per month	4 (2.5)	16 (7.3)		
Has used a contraceptive			2.31	0.13
No	7 (1.3)	18 (8.2)		
Yes	155 (95.7)	201 (91.8)		
Has had an abortion			2.68	0.10
No	81 (50.0)	128 (58.4)		
Yes	81 (50.0)	91 (41.6)		
Interval between last two abortions (n = 172, months)			0.89	0.64
< 6 months	12 (12.9)	11 (13.9)		
6–11 months	12 (12.9)	14 (17.7)		
≥ 12 months	69 (74.2)	54 (68.4)		
Number of children			20.87	0.01**
0	78 (48.1)	133 (60.7)		
1	43 (26.5)	68 (31.1)		
≥ 2	41 (25.3)	18 (8.2)		
Expected timing of next pregnancy (years)			11.83	0.01**
< 2 years	54 (33.3)	106 (48.4)		
≥ 2 years	56 (34.6)	62 (28.3)		
Does not want more children	36 (22.2)	27 (12.3)		
Unknown	16 (9.9)	24 (11.0)		
Aware of LARC			0.01	0.93
Yes	144 (88.9)	194 (88.6)		
No	18 (11.1)	25 (11.4)		
Previous LARC use			7.63	0.01**
Yes	21 (13.0)	11 (5.0)		
No	141 (87.0)	208 (95.0)		

*statistically significant by χ ^*2*^ *or t tests with p < 0.05*

** statistically significant by χ ^*2*^ *or t test with p < 0.01*

Variables that had significant correlation with a woman’s intention to use a LARC were retained in a subsequent logistic regression model. A best-fit regression model indicated that marital status, intercourse frequency, number of live children, planned timing of next pregnancy and previous LARC use were all significant predictors of a woman’s intention to use a LARC during the post-abortion period (Table 4).

**Table 4:** Factors associated with an intention to use LARCs among women with unintended pregnancies seeking abortions (*n* = 381)

Socio-demographic characteristics	Intended to use LARC	
	*OR (95% CI)*	*P*
Marital status		
Married	1.57 (1.00–2.47)	0.05
Single	1	
Frequency of sexual activity		
At least once per week	3.34 (1.03–10.78)	0.04*
< 4 times per month	2.34 (0.71–7.71)	0.16
< 1 time per month	1	
Number of children		
0	0.29 (0.13–0.68)	0.01**
1	0.32 (0.15–0.47)	0.01**
≥ 2	1	
Expected timing of next pregnancy (years)		
< 2 years	0.97 (0.43–2.12)	0.94
≥ 2 years	1.79 (0.80–4.01)	0.15
Does not want more children	1.18 (0.48–2.88)	1.18
Unknown	1	
Previous LARC use		
Yes	2.41 (1.06–5.47)	0.04*
No	1	

*statistically significant AOR with *p* < 0.05

**statistically significant AOR with *p* < 0.01

### Barriers to the use of LARCs during the immediate post-abortion period

Participants without intention to use a LARC during the post-abortion period (*n* = 219) were asked to report their perceived barriers to the use of LARCs. The barriers among this group varied. The most frequently mentioned barrier was “worry about future fertility” (56.2%). Additionally, participants reported the following: “fear of harm to health” (45.2%), “concern about irregular bleeding or spotting” (44.6%), “anxiety about IUD-related reproductive tract infections, movement, or loss of the device” (44.3%), “lack of awareness about LARC”, “fear of pain” (33.3%), and “the process to insert or remove IUDs is tedious” (23.7%; Table 5).

**Table 5:** Potential barriers among women who do not intend to use LARC during the post-abortion period (*n* = 219)

Women’s perceptions	*f*(%)	Rank by frequency
Worry about future fertility	123 (56.2)	1
Fear of harm to health	99 (45.2)	2
Worried about irregular bleeding or spotting	97 (44.3)	3
Fear of complications such as pregnancy with IUDs, reproductive tract infections, movement, or loss from uterus	91 (41.6)	4
Lack of awareness about LARC	79 (36.1)	5
Fear of pain	73 (33.3)	6
Process to insert or remove IUDs is tedious^a^	52 (23.7)	8
Fear of the surgical process of insertion or removal	51 (23.3)	7
Might cause abnormal menstruation	48 (21.9)	8
Performance without hormones	45 (20.5)	9
Friends or relatives have had bad experiences with LARC	26 (11.9)	10
Worried about weight gain	17 (7.8)	11
Too expensive	10 (4.6)	12
Have not been in a long-term relationship	9 (4.1)	13
Partner opposed to it	9 (4.1)	13
Prevented by religion from using the method	5 (2.3)	14
Have another satisfying method of contraception	3 (1.4)	15
Ineligible for the method because of being single	3 (1.4)	15
Unsure of future family plan	2 (0.9)	16

^a^For instance, if a Procreation Certificate is needed, then one must go to a health care provider to have the device inserted or removed

## Discussion

This study identified that only around two-fifths of Chinese women seeking abortion were interested in using a LARC. One-third intended to use IUDs, while only one in seven intended to use the implant method during the post-abortion period. These findings were consistent with a previous study conducted in China [26], which suggested that consultation may enhance access to LARCs. Wei and Liu [26] found that only 11.1 and 1.8% of women seeking abortion chose IUDs and contraceptive implants during the immediate post-abortion period prior to consultation with health professionals. After consultation, the intended use of IUDs and implants increased to 19.7 and 2.7%, respectively.

The findings of the present study also indicated that women seeking abortion preferred IUDs to contraceptive implants. The one-child policy was introduced to control population in mainland China in 1979 [27]; however, since 2016, women in mainland China have been allowed to give birth to two children. During the period in which the one-Child policy was enforced, immediate post-placental insertion of an IUD was practiced as part of the standard care package received by all women following the birth of their first child [28]. This may have led to a high general level of acceptance of IUDs in China, while implants were relatively less known or under-used [26, 29].

Frequency of intercourse, marital status, number of living children, previous LARC use and excepted timing of next pregnancy were all shown to be significant predictors of a woman’s intention to use a LARC. In contemporary mainland China, having sex before marriage is widely accepted among unmarried Chinese women, and this cohort showed a high intercourse frequency, as well as a high rate of induced abortion [30]. Research has also shown that the rate of IUD use among unmarried women is low (6304, 7.42%) [31] and that the majority of these women use short-acting contraceptives after abortion instead of LARCs [12]. However, post-placental insertion of an IUD is usually recommended to married women by health professionals [32]. The use of LARCs during the immediate post-abortion period is not the standard form of care. This indicates that LARCs might be under-utilized among unmarried women who have a high frequency of intercourse, and it is therefore important to address the barriers to LARC usage among unmarried women with high intercourse frequency and to promote its use among this group in order to reduce the risk of unintended pregnancy and repeated induced abortion.

Furthermore, the present study found that women with no or only one child, or planned to have a child within two years were found to have less intent to use a LARC. A previous study by Fleming and Sokoloff also showed that women with more than one child were more likely to be interested in using IUDs than nulliparous women [22]. A study conducted by Anguzu and Tweheyo similarly indicated that women who had living children were 1.5-times more likely to use a LARC compared to nulliparous women [20].

Previous LARC use was associated with whether participants were interested in LARC use in the post-abortion period. Evidence indicated that LARC users had a high degree of satisfaction with respect to this type of contraceptive [33]. Women who had previously used LARCs were 1.49-times more likely to intend to use a LARC [34].

Finally, this study indicated that barriers to the use of LARCs during the immediate post-abortion period included “worry about future fertility”, “fear of harm to health”, “may cause irregular bleeding”, “fear of complications”, and “lack of awareness about the methods”. A previous study indicated that because women believed that LARC use would negatively impact upon their future fertility, they did not choose a LARC [35]. We similarly found that worry about future fertility was the main reason that women seeking abortion did not intend to use a LARC in the immediate post-abortion period. It is obvious that worries about the risk and side effects of LARCs was one of the main reasons preventing women from using LARCs. A study that surveyed women aged 16 to 25 years showed that factors influencing contraceptive technology choices included “familiarity with the methods”, “whether or not the method contained hormones”, “likely effect on periods”, and “other side effects” [36]. A population of women who desired sterilization also had concerns about the side effects of LARCs [21].

Another barrier was a lack of awareness with regards to these methods. An internet-based study of 1982 female undergraduates at a large mid-western university in the United States found that lack of education about LARCs was the primary reason participants rejected this method [37]. Whitaker et al. [23] found that women aged 14–24 were likely to have positive attitudes around IUD methods after undergoing a three-minute educational intervention, suggesting that such interventions may improve positive attitudes towards LARC use among women seeking abortions [9]. There remain many myths regarding the use of LARCs, particularly regarding its potential effect on future fertility, which may prevent women of reproductive age using it to avoid unwanted pregnancy [38]. In order to help women to make informed decisions, health care professionals should pay significant attention to providing women with information relating to LARCs, especially with regards to alleviating the barriers.

Our study has some limitations which should be taken into consideration. Firstly, this investigation was conducted in only one hospital and most participants were employed women of Han ancestry. These findings may not therefore be representative of other settings or apply to those who are not of Han ancestry or are unemployed. In addition, this study relied upon a questionnaire that relied upon self-reporting by the respondents. Thus, there was the potential for recall bias in these data. Finally, adding qualitative study questions could provide additional insights into these findings in future research.

## Conclusions

This study has significant clinical utility for health care professionals working with Chinese women seeking abortion. To identify women seeking abortion who intend not to use LARCs, health care professionals should asses their marital status, frequency of sexual activity, number of children, planned timing of the next pregnancy, and previous LARC use.

Impaired future fertility, being harmful to health, irregular bleeding risk of IUD failure and lack of awareness with regards to LARCs were the main barriers to LARC use. It is important to promote the use of LARCs among unmarried Chinese women; particularly during the post-abortion period to reduce the risk of unintended pregnancy and repeated induced abortion. Health care professionals could develop strategies to enhance women’s intentions to use LARCs, such as providing information to alleviate the barriers to LARC use.
